# GaN/ZnO hybrid nanostructures for improved photocatalytic performance: One-step synthesis

**DOI:** 10.55730/1300-0527.3546

**Published:** 2023-02-02

**Authors:** Tugay ÜSTÜN, Bircan HASPULAT TAYMAZ, Volkan ESKİZEYBEK, Handan KAMIŞ, Ahmet AVCI

**Affiliations:** 1 Kahramankazan Vocational School, Başkent University, Ankara, Turkiye; 2 Department of Chemical Engineering, Faculty of Engineering and Natural Sciences, Konya Technical University, Konya, Turkiye; 3 Department of Materials Science and Engineering, Faculty of Engineering, Çanakkale Onsekiz Mart University, Çanakkale, Turkiye; 4 Department of Biomedical Engineering, Faculty of Engineering, Necmettin Erbakan University, Konya, Turkiye

**Keywords:** Hybrid materials, nanostructures, photocatalysts, malachite green

## Abstract

Nanostructured semiconductor materials are considered potential candidates for the degradation of textile wastewater via the photocatalytic process. This study aims to produce hexagonal gallium nitride (GaN) nanoplates and zinc oxide (ZnO) nanoparticles in a deionized water environment utilizing a one-step arc discharge process. Detailed characterization of samples has been completed via scanning electron microscope (SEM), transmission electron microscope (TEM), X-ray diffraction (XRD), Fourier transform infrared spectroscopy (FTIR), and UV visible spectroscopy methods. The hybrid nanostructure morphologies consist of nanoplates and nanorods of different sizes. The photoperformance of GaN/ZnO hybrid nanostructures was assessed via the malachite green (MG) dye degradation under UV exposure. Under UV exposure, the degradation yield reached 98% in 60 min. Compared to individual ZnO and GaN nanoparticles, the photocatalytic reaction rate of the GaN/ZnO photocatalyst is 2.2 and 3.6 times faster, respectively. Besides, the GaN/ZnO hybrid nanostructures show excellent photocatalytic stability. The energy consumption of the photocatalytic degradation in the presence of GaN/ZnO hybrid nanostructures was 1.688 kWhL^−1^. These results demonstrate that the GaN/ZnO hybrid nanostructures with improved photocatalytic activity are a reasonable option for the decomposition of textile wastewater under UV light exposure.

## 1. Introduction

The development of nanostructures with designed complex morphologies and compositions offers new potential for improving the properties of the neat structure by the synergistic combination. Due to their potential for several functionalities, such as catalytic, magnetic, and optoelectronic ones, nanoparticle hybridization has gained considerable attention lately [1]. For instance, electrical conduction can be increased by incorporating various metal and metal oxide nanoparticles into graphene [2]. The hybridization-developed features enable applications in various industries like electronics, sensors, and energy [3,4]. Recently, Wide-band-gap nanoscale semiconductors with superior carrier density and mobility can operate at high temperatures, and voltages have attracted much attention [5–9]. The III group-nitride semiconductor material GaN, which exhibits a wide bandgap at the nanoscale, is one of the most promising ones (3.4 eV at room temperature) [10]. Great success has already been achieved in many optoelectronic and electronic devices, including light-emitting diodes (LEDs) [11], solar cells [11], chemical sensors [12–14], and biosensors [15] with the implementation of GaN nanostructures. The wide-band-gap metal oxides can be used as a photocatalyst for decomposing dyes, organic molecules, and different contaminates, reducing CO_2_ and splitting water [16,17]. One of the most critical parameters in the photocatalytic degradation process is chemical stability due to prolonged contact time. To improve the chemical stability of metal oxides like ZnO and TiO_2_ can be coupled with GaN [18]. Photoconductive metal-oxide-semiconductors are attracted materials because of their active surface and UV sensing processes [19]. ZnO is a broadband gap material and holds a significant place within the semiconductor group (3.37 eV) [20]. This oxide creates potential applications in optoelectronics due to its physical properties [21,22]. ZnO nanostructures show excellent photocatalytic activity in reducing environmental pollution [23]. The direct energy gap of ZnO exhibits similar properties as in GaN, and it is expected to use ZnO in applications using GaN [24]. Therefore, it is also possible to use GaN and ZnO to improve the properties of materials [25]. The combination of GaN and ZnO nanostructures has led to lower band gaps (2.6–2.9 eV for containing a high amount of GaN) [26], allowing absorption of visible light and increasing the photocatalytic dye degradation process.

There are many methods for synthesizing GaN and ZnO nanoparticles with various morphologies, such as rods [23], plates [27], and wires [28], including the sol-gel technique [29], spray pyrolysis [30], chemical vapor deposition [31], cathodic electrodeposition [32], plasma pyrolysis, and chemical vapor synthesis [33]. The arc discharge (AD) method emerges as a cheap and straightforward process in a liquid medium to produce nanoparticles on a large scale [34].

The recent trend in the photocatalytic dye degradation process is to develop which can be degraded organic contaminants in a possibly shorter time. Van [35] investigated the photocatalytic performance of GaN-ZnO doped g-C_3_N_4_ by degradation methylene blue (MB) under visible light. The 50 mg/L MB dye completely degraded after 7 h illumination in the presence of 500 mg/L GaN-ZnO doped g-C_3_N_4_. In the other work, the GaN:ZnO NPs synthesized with the hydrothermal method and used as photocatalysts for degradation of 20 mg/L MB under visible light irradiation. The 20 mg/L MB dye was completely degraded after 100 min under visible light using GaN:ZnO [36].

This study aims to obtain the hybrid GaN and ZnO nanostructures using an in situ one-step synthesis method with enhanced photocatalytic performance by the synergic combination of the components. In this work, we have synthesized GaN/ZnO hybrid nanostructures by AD method in a deionized water medium. The characterization of GaN/ZnO nanostructures was investigated by SEM, EDX-mapping, TEM, XRD, FTIR, and UV-vis methods. Also, we employed the decomposition of MG under UV illumination to assess the photo performance of the obtained GaN/ZnO hybrid nanostructures. To understand the effect of the combination of GaN and ZnO NPs, the photocatalytic performance of pure GaN and pure ZnO NPs was investigated by the degradation of MG dye under UV light illumination. The one-step synthesis GaN/ZnO nanostructures show enhanced photocatalytic performance compared with the pure GaN and ZnO nanostructure, which indicates that the GaN/ZnO nanostructure is a promising candidate for photodegradation of organic pollutants.

## 2. Materials and methods

### 2.1. Materials

GaN powder from Merck and Zn rods from Alfa Aesar, both with a 99.99 % purity, were used to produce the hybrid GaN/ZnO nanostructures. To examine the photo performance of hybrid nanostructures, MG was acquired from Merck.

### 2.2. Synthesis of GaN/ZnO hybrid nanostructures

The arc discharge method, an efficient and inexpensive method, has been preferred in synthesizing GaN/ZnO hybrid nanostructures. In the arc discharge apparatus, zinc electrodes are used as an anode and cathode, and deionized water is used as a medium. A zinc rod (10 × 20 mm) served as the cathode. To ensure smooth arc production during the experiment, a smooth surface is constructed for the cathode rod. A second zinc rod (12 × 60 mm) serving as an anode was bored and 0.2 g of GaN powder was introduced to the rod. Touching the anode to the cathode started an arc discharge in the deionized water. To achieve a consistent arc, the discharge voltage must be maintained between 20 and 30 V. The current (50 A), which has been obtained through several experimental tests, is the perfect value for producing nanoparticles with optimal morphologies in large quantities [37]. Arc discharge persisted until the anode electrode’s GaN powder was depleted. The resulting powders were collected in a beaker and vacuum-dried at 80 °C.

### 2.3. Characterization

For analyzing the morphology of the synthesized materials, a JEOL/JSM-6335F-EDS SEM was utilized. TEM image was captured via a JEOL 2100 transmission electron microscope working at 300 kV. Cu Ka radiation (k = 0.15418 nm) was used to create the X-ray diffraction (XRD) pattern, which was then recorded using a Shimadzu XRD-6000 X-ray diffractometer working at 40 kV and 30 mA. The HR4000 UV-VIS from Ocean Optics was used to acquire UV-VIS spectra. The FTIR spectrum was recorded via Perkin Elmer Spectrum 100.

### 2.4. Measurement of photocatalytic activity

The photocatalytic decomposition of MG was achieved in a photoreactor (Luzchem Research Inc., Canada) accoutered with 8 UVC bulbs with emission at = 254 nm. A digital lux meter recorded 946 lx as the UV light’s intensity. In a quartz tube, 0.6 mg of the nanoparticle and 3 mL of MG aqueous solution with a concentration of 1.5 × 10^−5^ M were added. To achieve the adsorption-desorption equilibrium, this dye-photocatalyst mixture was magnetically agitated for 30 min in the dark. The photo decolorization of MG then proceeded after the lamps were switched on at room temperature. The UV-vis spectroscopy technique was used to determine the MG dye’s concentration. Equation 1 is used to obtain the dye degradation efficiency.


(Eq. 1) $$Degradation\;(\%)=\frac{C_{0}-C}{C_{0}}\times 100$$

where *C_0_* denotes the initial dye concentration and *C* denotes the dye concentration after a certain irradiation time.

To understand the photocatalytic stability performance, GaN/ZnO hybrid nanostructures were utilized for five photocatalytic cycles. After the first photocatalytic experiment, the catalyst was collected from suspension with centrifugation and decantation. After, the photocatalyst was introduced to a new dye solution the experiment procedure was repeated five times.

### 2.5. Energy consumption of photocatalytic dye degradation

The electrical energy per order (EEO) was calculated to determine the energy consumption of photocatalytic MG degradation. E_EO_ was defined based on the electrical energy required for degradation of MG in the presence of GaN/ZnO hybrid nanostructure, pure ZnO, and pure GaN after 60 min UV light exposure as follows:


(Eq. 2) $$E_{EO}=\frac{P\times t}{V\times \text{log }(\frac{C_{0}}{C_{t}})}$$

Where P is the power (kW) of the lamps, V is the volume (L) of degraded dye in the time t (h), and C_0_ and C_t_ are the initial and final dye concentrations, respectively.

## 3. Result and discussion

### 3.1. Structure and morphology

**Figure 1:**
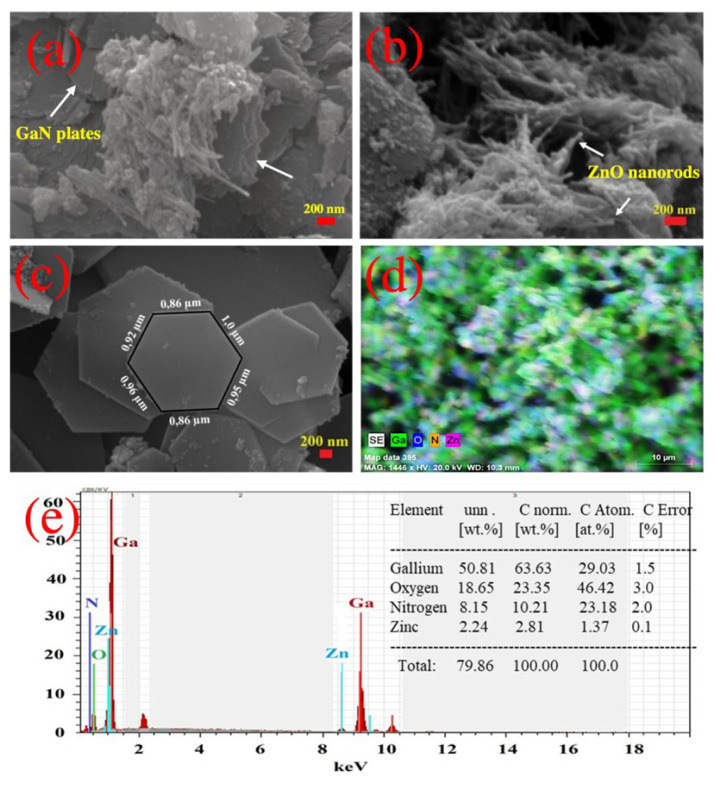
(a–c) SEM images of the GaN/ZnO hybrid structures at different regions, (d) EDS mapping of the hybrid structure (e) EDS spectrum of the GaN/ZnO hybrid nanostructures.

Figures 1a–1c show the SEM images of the GaN/ZnO hybrid nanostructures produced via the AD method from different regions. Two distinct morphologies, hexagonal-shaped plates (2D) and nanorods (1D) with different sizes, are observed. ZnO nanorods are regarded as an agglomerated structure with approximately 10–30 nm diameters and in a micron length on GaN nanoparticles (Figures 1a–1b). Our previous studies [20,38] showed that the ZnO nanostructure synthesized by AD methods displayed similar nanorod structure morphology. The GaN nanoplates are displayed with dimensions ranging from one to a few microns, and their thickness is approximately 50–150 nm (Figure 1c). The produced plates are exactly hexagonal, and in certain places, these plates are interlinked, as represented previously by [27]. The EDS mapping represents gallium, oxygen, nitrogen, and zinc elements with a homogeneous distribution in the hybrid nanostructure (Figure 1d). The EDS spectrum is given in Figure 1e, and the chemical composition of the synthesized hybrid nanostructures shows no additional peaks, indicating high purity (Figure 1e). Besides, since the amount of gallium is higher than zinc, GaN nanoplates can be regarded as an abundant structure in the hybrid structure.

**Figure 2:**
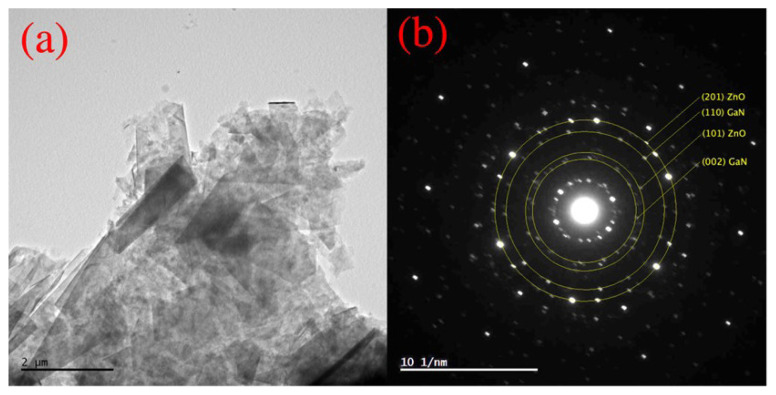
(a) TEM image and (b) SAED image of GaN/ZnO hybrid nanostructures.

TEM image of agglomerated GaN/ZnO hybrid nanostructures is given in Figure 2a. Two different morphologies are identified in the synthesized GaN/ZnO hybrid nanostructures (Figure 2a). GaN nanoplates are observed with widths varying from 1 to a few μm. As observed with SEM analysis, ZnO nanorods mainly represent an agglomerated structure GaN plates. The SAED analysis of GaN/ZnO hybrid nanostructures indicates is given in Figure 2b. These rings are attributed to a polycrystalline structure (Figure 2b). The interplanar distances are calculated as 0.265, 0.2448, 0.150, and 0.130 nm using the image processing method (ImageJ, https://imagej.nih.gov/ij/), which are in good agreement with (002) plane of GaN, (101) plane of ZnO, (110) plane of GaN and (201) plane of ZnO, respectively.

**Figure 3:**
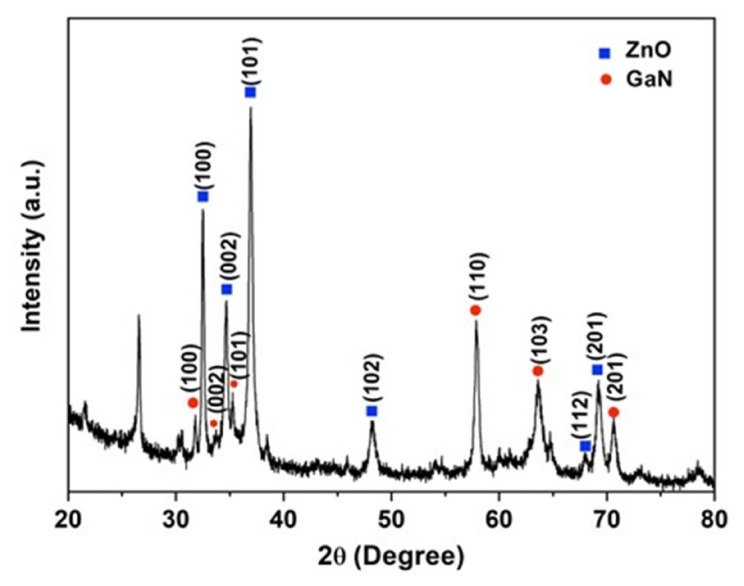
The XRD pattern of the GaN/ZnO hybrid nanostructures.

The XRD pattern of the synthesized GaN/ZnO hybrid nanostructures is given in Figure 3. As a result of phase identification, the coexistence of wurtzite ZnO and wurtzite GaN phases is revealed (Figure 3). The hexagonal wurtzite structure of the indicated ZnO peaks possesses lattice parameters a = 0.324 nm and c = 0.519 nm (JCPDS No. 30–1451). According to Scherrer’s formula, which is illustrated in Equation 3, the average particle size of the ZnO phase is roughly 30 nm.


(Eq. 3) $$D=\frac{K\lambda }{\beta cos\theta }$$

Here K = 0.9 is a dimensionless shape factor, *λ* (nm) indicates the wavelength of CuKα radiation (1.5418 Å), *θ* is the Bragg angle and *β* means the FWHM of the diffraction peak. The XRD pattern shows that the ZnO nanoparticles have a strongly oriented shape. The three most noticeable diffraction peaks, (100), (002), and (101), are all consistent with hexagonal wurtzite GaN (JCPDS No. 88–2361), with a and c wavelengths of 0.316 and 0.512 nm, respectively, since the GaN phase is abundant in the hybrid structure as confirmed by EDS analysis.

**Figure 4:**
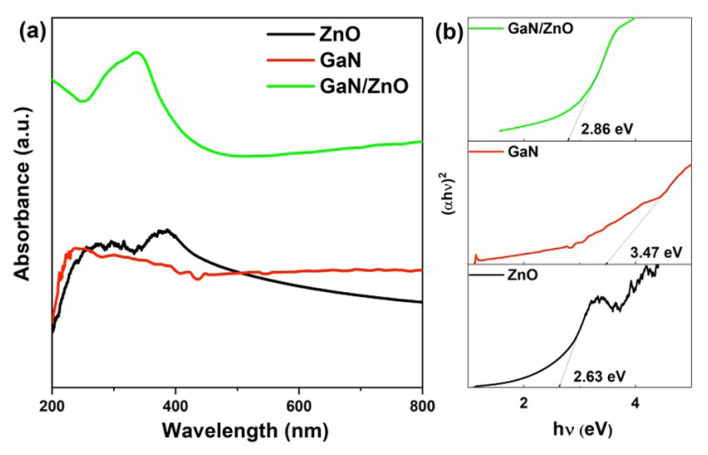
(a) The UV-vis spectrum and (b) the band gap estimation of pure ZnO, pure GaN, and GaN/ZnO hybrid nanostructures.

The absorbance of pure ZnO, pure GaN, and GaN/ZnO hybrid nanostructures are displayed in Figure 4a from 200 to 800 nm. The absorbance spectrum of pure ZnO indicates two absorption bands at 292 and 383 nm. The GaN possesses an absorption band at 240 nm. The absorption spectrum of GaN/ZnO hybrid nanostructures represents a broad absorption band with two distinct shoulders centered at 335 and 310 nm, and a long adsorption tail into the visible light area (Figure 4a). The absorption spectrum of GaN/ZnO hybrid nanostructures represents a similar curve of both pure GaN and pure ZnO structures. The band at 383 nm belonging to ZnO shifted to a small wavelength due to the interaction between ZnO and GaN phases.

The band gap (Eg) of the GaN/ZnO hybrid nanostructures estimated Tauc Plots as (αhν)^n^ versus hν from the UV-vis spectrum, where *α* absorption coefficient, *h* Planck constant, light frequency, and *n* = 2 for the direct band gap material (Figure 4b) [39]. The band gap of pure ZnO, pure GaN, and GaN/ZnO hybrid nanostructures are estimated to be 2.63, 3.47, and 2.86 eV (Figure 4b).

**Figure 5:**
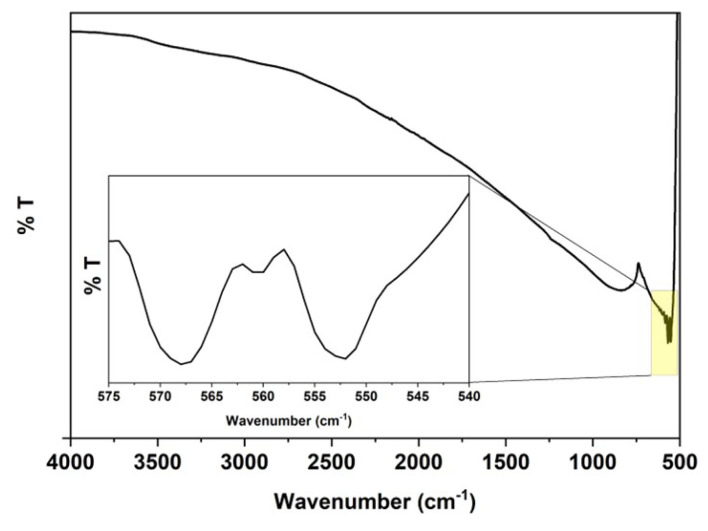
FTIR spectrum of GaN/ZnO hybrid nanostructure.

The FTIR spectrum of GaN/ZnO hybrid nanostructures is given in Figure 5. The absorption bands of 567.74 and 560.40 cm^−1^ correspond to Ga-N stretching vibration in hexagonal type and Ga-N stretching vibration peak [40]. The band at 546 cm^−1^ indicates the presence of Zn-O vibrations (Figure 5) [41].

### 3.2. Photo performance of GaN/ZnO hybrid nanostructures

The decomposition of the MG dye solution under an ultraviolet light source was utilized to assess the photo performance of GaN/ZnO hybrid nanostructures. To compare with the photo performance of GaN/ZnO hybrid nanostructures, the photocatalytic efficiency of pristine ZnO and pristine GaN nanostructures was also studied under the same circumstances.

**Figure 6:**
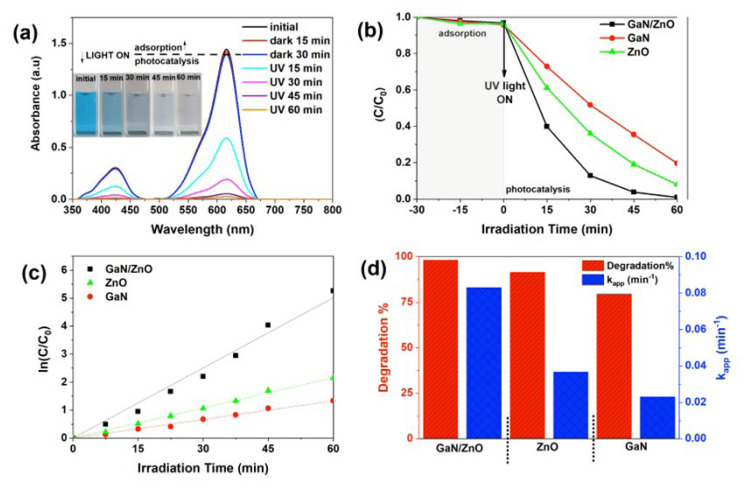
(a) The UV-vis absorbance spectra and inset color change of MG dye (inset figure 6a; photographs of decomposition comparison) comparison of (b) the apparent rate constants of the MG with respect to time intervals and (c–d) degradation efficiencies and k_app_ of the MG at 60 min in the presence GaN and ZnO nanostructures and GaN/ZnO hybrid nanostructures under UV light irradiation (0.2 mg/mL photocatalyts; concentration of dye: 1.5 × 10^−5^ M).

Negligible photocatalytic activities are indicated by blank tests, such as catalyst-free or light sources [38]. Figure 6a displays the variation in the MB dye’s adsorption spectra when GaN/ZnO hybrid nanostructures are present and UV light (photocatalysis) is irradiated for intervals of 15 min. The characteristic peaks of MG dye at 423 and 615 nm decrease suddenly after 15 min exposure under UV light illumination (Figure 6a). After 60 min of UV light exposure, the MG’s distinctive peaks almost completely vanished. The MG dye’s color change was also seen inset Figure 6a. The comparison of the decolorization rate of MG in the presence of GaN/ZnO hybrid nanostructures, pure ZnO and pure GaN under UV light and dark conditions (Figures 6b, 6c). It can be observed clearly that the degradation of MG dye reached around 60% in the presence of GaN/ZnO hybrid nanostructures under 15 min UV light exposure. Also, the MG dye has been degraded nearly completely by GaN/ZnO hybrid nanostructures after 60 min. In the same conditions, the decolorization efficiency are 79% and 91% under UV light irradiation in the presence of the pure GaN and pure ZnO nanostructure. In the dark condition, the decolorization of MG dye is around only 5% with adsorption process. Also, the reaction rate constant of photocatalysts were determined via the pseudo first-order kinetic model. The pseudo first-order kinetic has been widely used to model the photocatalytic process and the reaction rate can be applied as Equation 4,


(Eq. 4) $$-ln\frac{C}{C_{0}}=kt$$

where C is the measured MG concentration at different intervals, C_0_ is the initial MG dye concentration measured after 60 min absorption and k refers to the reaction rate kinetic constant, and t is the irradiation time. The reaction rate constant k of the GaN/ZnO catalyst is 0.08899 min^−1^ which is approximately 4 and 3 times higher than the reaction rate constant pure GaN and pure ZnO catalysts under UV light irradiation, respectively. The results indicate that the GaN/ZnO hybrid nanostructure shows superior photocatalytic activity for decolorization MG dye under UV light irradiation. Also, GaN/ZnO hybrid nanostructures indicate excellent photocatalytic activity when compared with pure GaN and pure ZnO catalysts. The Figure 6d is given to compare the degradation efficiency of MG dye and the photocatalytic reaction rate under 60 min UV light irradiation in the presence of GaN/ZnO hybrid nanostructures and GaN and ZnO nanostructures (Figure 6d). The results are given in Table 1.

**Figure 7:**
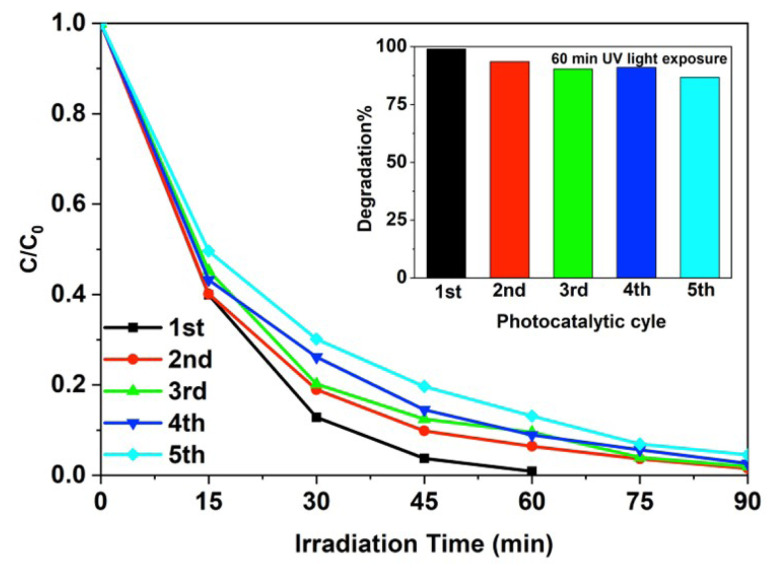
The photocatalytic stability of GaN/ZnO for the degradation of MG under UV light and inset figure: the comparison of the degradation efficiency for the 60 min UV light illumination of MG dye (0.2 mg/mL photocatalysts; concentration of dye: 1.5 × 10^−5^ M).

The photocatalytic stability of GaN/ZnO hybrid nanostructure was investigated with the five photocatalytic dye degradation processes. For each photocatalytic usage, GaN/ZnO was collected with centrifugation and then added to a new dye solution. After first photocatalytic cycle, the photocatalytic activity of GaN/ZnO was decreased. MG dye decolorized in 90 min under UV light irradiation in the second photocatalytic usage. No apparent deactivation of GaN/ZnO was observed after the second usage under UV light irradiation for degradation of MG dye (Figure 7).

The comparison between this work and the data reported in other literature is presented in Table 2. The synthesized GaN/ZnO photocatalyst is a potential and alternative candidate to use in photocatalytic wastewater treatment.

### 3.3. Energy consumption

The electrical energy consumption of photocatalytic degradation of MG was calculated. The E_EO_ values were 1.688, 2.51, and 3.48 kWhL^−1^ for photocatalytic degradation of MG in the presence of GaN/ZnO hybrid nanostructure, pure ZnO, and pure GaN under UV light illumination. The photocatalytic degradation of MG in the presence of GaN/ZnO hybrid nanostructure needs the lowest energy compared with the pure ZnO and pure GaN.

The energy demand in the presence of pure ZnO and pure GaN of photocatalytic degradation is around 2 times higher than that of GaN/ZnO hybrid nanostructure. The results show that GaN/ZnO hybrid nanostructure is an energy saver photocatalyst than pure ZnO and pure GaN for the degradation of MG under UV light illumination.

## 4. Conclusion

The GaN/ZnO hybrid nanostructures were synthesized via the AD method. The morphology of GaN/ZnO consists of hexagonal nanoplate and nanorod structures. The EDS analysis image and spectrum showed no additional peaks, excluding gallium, oxygen, nitrogen, and zinc elements. Due to the synergistic effect, the band gap of the GaN/ZnO hybrid nanostructures was predicted to be 2.86 eV. By degrading the MG dye solution under a UV light source, the photo performance of GaN/ZnO hybrid nanostructures was investigated. To compare with the photo performance of GaN/ZnO hybrid nanostructures, the photocatalytic activities of pure ZnO and pure GaN nanostructures were also studied under similar circumstances. After 60 min of UV light exposure, GaN/ZnO hybrid nanostructures had totally decomposed the MG dye. Under similar circumstances, and in the presence of pure GaN and pure ZnO nanostructures, the decolorization efficiencies are 79% and 91%, respectively. Also, the GaN/ZnO hybrid nanostructure exhibits excellent photocatalytic stability. In the fifth photocatalytic cycle, the MG dye was completely decomposed in the presence of the GaN/ZnO hybrid nanostructure under UV light illumination. Moreover, GaN/ZnO hybrid nanostructure is a more energy saver photocatalyst than pure ZnO and pure GaN for the degradation of MG under UV light illumination. GaN/ZnO hybrid nanostructures are anticipated to be a strong contender to be used as a high-performance photocatalyst for the oxidation of organic dyes when exposed to UV radiation.

## Figures and Tables

**Table 1:** The comparison degradation efficiencies and *k* of MG in the presence of GaN/ZnO hybrid nanostructures and GaN and ZnO nanostructures under UV exposure after 60 min (0.2 mg/mL photocatalyst; initial concentration of dye: 1.5 × 10^−5^ M )

	Degradation %	k_app_ (min^−1^)	R^2^
GaN/ZnO	98	0.08889	0.9882
GaN	79	0.02307	0.9992
ZnO	91	0.03671	0.9987

**Table 2:** Comparison of previously reported photocatalysts with degradation efficiency.

Photocatalyst	Pollution	Light	%Degradation	Time (min)	Reference
GaN-ZnO/g-C_3_N_4_	MB	UV	98.1	300	[35]
MOF-derived C-ZnO/PVDF	MB	UV	95%	270	[42]
Mn_3_O_4_@ZnO	MG	UV	89.99	240	[43]
SnO_2_/ZnO	MG	Xenon Lamp	98	100	[44]
GaN:ZnO	MB	Visible	100	100	[36]
GaN/ZnO	MG	UV	98	90	This work
